# Retraction: Lipoprotein Ratios: Correlation With Thyroid Profile and Glycated Hemoglobin (HbA1c) Among Diabetes Mellitus Patients

**DOI:** 10.7759/cureus.r158

**Published:** 2025-01-10

**Authors:** Ayan Banerjee, Jagriti LNU, Prabhat LNU, Akash Bansal

**Affiliations:** 1 Biochemistry, All India Institute of Medical Sciences, Patna, Patna, IND; 2 Biochemistry, All India Institute of Medical Sciences, Gorakhpur, Gorakhpur, IND

This article has been retracted by the Editors-in-Chief at the request of the authors due to concerns regarding adherence to ethics policies during data collection. The Editors-in-Chief have reviewed these concerns and made the decision to retract this article as the integrity of the study and results are now in question.

